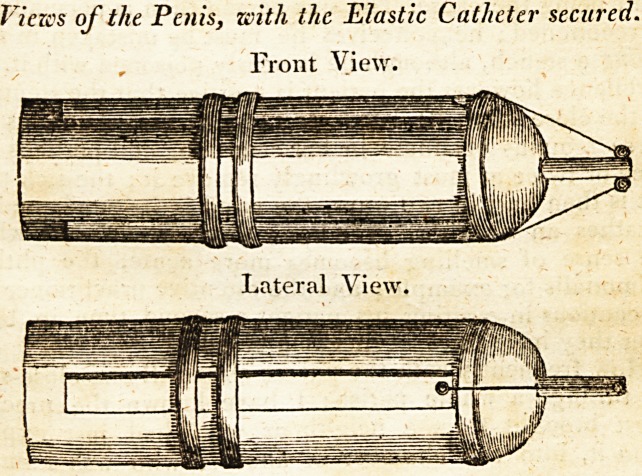# To the Editors of the Medical and Physical Journal

**Published:** 1803-08-01

**Authors:** 


### *To the Editors of the Medical and Physical Journal.*

GENTLEMEN,

THE papers I have had the honour of addressing to you on the furniture of a sick chamber, and hints respecting variolous infection, partook, I am aware, too much of the diffusive turn of a young author, and as such I crave indulgence; you will now find them stripped of their drapery, and the reader will arrive at the information without his route being very circuitous; and if you think the following also deserving the public attention, you will please to insert it.

There are certain complaints of the urethra and neck of the bladder, that require, in my opinion, the constant abidement of the elastic catheter in the urinary passage during the continuance of the disease. I do not mean that the same catheter must remain the whole time, as calculous incrustations would undoubtedly take place, and a change of the instrument, to prevent such an unpleasant event, must be frequently necessary.

As I have not frequent leisure to refer to the different authors on surgery, it was imprinted on my mind, that the plan I had proposed for securing the elastic catheter in the bladder, and which was adopted in a consultation a few years back, had novelty in it; in short, its simplicity had led me to believe that it must be pretty generally practised, till I purchased Mr. Hey's cases.* In a work of such respectability, I found a method recommended very different from mine, and, as I think, inferior to it.

The plan I recommend is this, and the following drawings will more fully illustrate it. The elastic catheter is to be passed in the usual way; and when it is in the bladder, the patient, or assistant, is to press the catheter rather farther in, than where it would remain without pressure. We are then to apply a slip of adhesive plaster on each side the penis, nearly its whole length, and to the end of each plaster, near the preputium, a tape is to be fixed. These are to be tied to the rings of the catheter; and a little more than midway

* The mention of Mr. Hey's name, points out the very great utility of your Journal, for had there been a respectable publication, like yours, many years ago, so much valuable treasure as his work contains, would not in all probability have been locked up.

midway of the penis, you are to encircle it and the two lateral plasters, with two other slips of plaster, taking care these surrounding plasters are not placed on each other, and this is all that is necessary.

My motive for pressing the catheter rather more into the bladder, is to allow of the swelling of the penis, which must sometimes happen, particularly in young persons, during sleep.

The circular plasters are intended to confine the lateral ones; and my reason for using two, is, in case one should give way.

Cleanliness may call for a frequent renewal of the plasters, and, if necessary, to wear a T bandage for any external opening in *perinæo,* or other cause, it need not interfere with the plasters.

As to the protection of the end of the catheter, I have ever found the patient's contrivances the best; do not put him in a state of security as to this particular, by any thing you do; leave this part of the business to the patient, and he will attentively, and safely, guard it from harm and inconvenience.

## Figures and Tables

**Figure f1:**